# Identifying significant temporal variation in time course microarray data without replicates

**DOI:** 10.1186/1471-2105-10-96

**Published:** 2009-03-26

**Authors:** Stephen C Billups, Margaret C Neville, Michael Rudolph, Weston Porter, Pepper Schedin

**Affiliations:** 1 Department of Mathematical and Statistical Sciences, University of Colorado, Denver, Colorado, USA; 2 Department of Physiology, University of Colorado, Aurora, Colorado, USA; 3 Department of Pathology, University of Colorado, Aurora, Colorado, USA; 4 Department of Interactive Biosciences, Texas A & M University, College Station, Texas, USA; 5 Department of Medicine, Colorado Cancer Center, AMC Cancer Research Center, University of Colorado, Aurora, Colorado, USA

## Abstract

**Background:**

An important component of time course microarray studies is the identification of genes that demonstrate significant time-dependent variation in their expression levels. Until recently, available methods for performing such significance tests required replicates of individual time points. This paper describes a replicate-free method that was developed as part of a study of the estrous cycle in the rat mammary gland in which no replicate data was collected.

**Results:**

A temporal test statistic is proposed that is based on the degree to which data are smoothed when fit by a spline function. An algorithm is presented that uses this test statistic together with a false discovery rate method to identify genes whose expression profiles exhibit significant temporal variation. The algorithm is tested on simulated data, and is compared with another recently published replicate-free method. The simulated data consists both of genes with known temporal dependencies, and genes from a null distribution. The proposed algorithm identifies a larger percentage of the time-dependent genes for a given false discovery rate. Use of the algorithm in a study of the estrous cycle in the rat mammary gland resulted in the identification of genes exhibiting distinct circadian variation. These results were confirmed in follow-up laboratory experiments.

**Conclusion:**

The proposed algorithm provides a new approach for identifying expression profiles with significant temporal variation without relying on replicates. When compared with a recently published algorithm on simulated data, the proposed algorithm appears to identify a larger percentage of time-dependent genes for a given false discovery rate. The development of the algorithm was instrumental in revealing the presence of circadian variation in the virgin rat mammary gland during the estrous cycle.

## Background

In recent years, there has been considerable interest in using gene expression microarray data to study the dynamic behavior of cells. Microarrays allow researchers to take a "snapshot" of the state of a cell by measuring the mRNA expression levels of thousands of genes simultaneously. By taking multiple such "snapshots" at different times, one gains a dynamic picture of how expression levels change over time. A review article by Bar-Joseph [1] gives an excellent summary of many of the issues involved and methods developed for analyzing time course microarray data. Early analysis techniques applied methods that were originally designed for analyzing static data. But there are important differences between static experiments and time course experiments, which have motivated the development of specialized methods for analyzing time course data. In static experiments, data are collected for a number of different experimental conditions. There may be little or no mathematical relationship between these conditions, so they are usually represented as categorical data. In such experiments, it is essential to have several replicates from each condition. In time course experiments, time is a quantitative variable, so the order of the data and the spacing between time points matters. This difference can be exploited to develop more powerful techniques for analyzing time course data. Moreover, there is no longer an inherent requirement for replicates.

Based on this insight, we designed a microarray study of the estrous cycle of the rat mammary gland in which microarray data were collected at distinct time points without replicates. As part of this study, we developed an algorithm for identifying genes with significant temporal variation that does not rely on replicates. The algorithm is based on fitting the data with B-splines, which has a smoothing effect. A test statistic was developed that measures the magnitude of this smoothing effect relative to the overall variation in the data. This test statistic is then used in a false discovery rate procedure to identify significant genes.

The method described here, which we shall refer to as *Method 1*, has some notable similarities with a method recently proposed in [2], which we shall call *Method 2*. Both methods involve fitting the data with a B-spline model and neither method requires replicates. But there are some important differences between the two methods. First, Method 2 is based upon generalizing traditional analysis of variance methodologies to time series analysis. In contrast, Method 1 is motivated by the smoothing effect resulting from fitting the data by a spline function. It is designed explicitly to identify lower frequency variation in the data, which is not affected as much by the smoothing. Another difference is the technique used to assess statistical significance. In Method 2, a bootstrapping method is used to estimate a null distribution of the test statistic for each gene. In contrast, our method estimates a null distribution by recalculating the test statistic on a permuted data set.

## Results

### Algorithm description

To describe our algorithm, we assume that gene expression data were collected at *T *time points denoted *t*_1_,..., *t*_*T*_, given in nondecreasing order (i.e., *t*_1 _≤ *t*_2 _≤ ... ≤ *t*_*T*_). Letting *G *denote the number of genes, *Y *denotes the *G *× *T *array whose *ij*th entry *Y*_*ij *_is the normalized log-expression level of the *i*th gene at time point *t*_*j*_. *Y*_*i *_denotes the *i*th row of *Y*, which will be referred to as the *log-expression profile *of gene *i*.

Conceptually, we can view the data as being comprised of both time-dependent and time-independent variation. Specifically, we assume the following model of the data:

*Y*_*ij *_= *f*_*i*_(*t*_*j*_) + ϵ_*ij*_

where *f*_*i*_(*t*) is a continuous function of time, and ϵ_*ij *_represents the time-independent variability, which may be due to noise, or to other influences such as sample-to-sample heterogeneity.

The function *f*_*i*_(*t*) summarizes the influences of many different time-dependent biological processes, such as cell-cycles, circadian rhythms, development patterns, hormonal fluctuations, etc. Any such influences that occur at too high a frequency (relative to the sampling rate) cannot be distinguished from noise. So we assume the biologist is only interested in detecting the low frequency variation in *f*_*i*_.

With this in mind, we loosely define a gene to exhibit *significant temporal variation *if its expression profile exhibits "significant" low frequency variation. To translate this concept into a statistical test, we fit the expression profile of each gene with a "smooth" function of time *ϕ*(*t*) using B-splines.

#### Splines

Splines are piecewise polynomial functions, which are defined with respect to a non-decreasing set of *knots τ*_1 _≤ *τ*_2 _≤ ... ≤ *τ*_*M*+1_. Between two distinct consecutive breakpoints, the spline function is a polynomial of a specified degree. The order of the spline is defined to be one greater than the degrees of the polynomials. Typically, the interior knots are chosen to be distinct time points, whereas for technical reasons, the first and last knots are repeated *K *times, where *K *is the order of the spline. For data approximation purposes, a convenient method for defining splines is to define a basis for the set of piecewise polynomial functions of a given order. A popular choice of basis is the *B-spline *basis, which is defined using the Cox-de Boor recursion formula [3] as follows:

(1)

In this formula, *k *represents the order of the spline. For notational convenience, we define *b*_*i*_(*t*) = *b*_*i*, *K*_(*t*). ℬ = {*b*_1_(*t*),..., *b*_*M*_(*t*)} is the basis for the set of splines of order *K*. Using this basis, it is possible to represent any *K*-order spline function *ϕ*(*t*) as a linear combination of the basis functions. That is,

(2)

where *c*_*m*_, *m *= 1,..., *M *are the spline coefficients which uniquely define *ϕ*(*t*).

This representation is particularly convenient for fitting splines to a set of data points. The goal is to determine for each gene *i *the coefficients *C*_*i *_= [*c*_1_,..., *c*_*M*_] which give the best approximation of the log-expression profile *Y*_*i *_in the least squares sense. The first step is to define the "spline collocation" matrix *S*, which stores the values of the basis functions evaluated at the sample time points. The entries of *S *are defined by *S*_*mj *_= *b*_*m*_(*t*_*j*_). The least-squares approximation is then calculated by the equation

(3) *C*_*i *_= *Y*_*i*_*S*^+^,

where *S*^+ ^denotes the Moore-Penrose pseudoinverse of *S *(see [4]).

#### Test statistic

The spline approximation described above smooths out rapid fluctuations in the log-expression levels of a gene, while preserving longer term trends. This observation motivates the definition of the following test statistic, which is inversely related to the magnitude of the smoothing effect:

(4)

where *Z*_*i *_:= [*ϕ*_*i*_(*t*_1_), *ϕ*_*i*_(*t*_2_),..., *ϕ*_*i*_(*t*_*n*_)] is the vector of interpolated function values, *ϕ*_*i*_(*t*) is the approximating spline function for gene *i*, and Var denotes the variance. Intuitively, a small value of *ρ *corresponds to a large smoothing effect, which suggests that any long-term temporal trends are small relative to the overall variation in the log-expression levels. In contrast, a large value of *ρ *indicates the presence of a meaningful temporal trend.

To determine which genes demonstrate significant temporal variation, we use a false discovery rate procedure in conjunction with the test statistic *ρ*. To apply this procedure, it is necessary to approximate an expected distribution for *ρ *under the assumption that there is no temporal variation. This is accomplished by creating a permuted data set , which is generated by reordering the columns of the original data matrix *Y *in such a way that columns that were originally close together become far apart in . To make this more precise, a *permutation vector π *is specified, which is simply a rearrangement of the integers 1,..., *T*. Then, define , where *π*_*j *_is the *j*th component of *π*.

The *ρ *statistic is calculated for each "gene" in this permuted data set. The resulting values are then sorted, yielding an estimated null distribution. The estimated distribution is used to calculate *p*-values for each gene as follows: for gene *i*, the *p*-value is calculated by



where *L*_*i *_is the number of *ρ *values from the permuted data set that are larger than *ρ*_*i*_. Finally, a false discovery rate procedure [5] is used to choose significant genes.

#### Choosing a permutation vector

The permutation vector *π *should be chosen so that columns that are close together in the original data matrix *Y *will be far apart in the permuted matrix . Because many biological processes are cyclic, we define the distance between two indices in a way that accounts for wrapping around. Specifically, we define

Δ(*i*, *j*) = min{|*i *- *j*|, |*n *+ *i *- *j*|, |*i *- *j *- *n*|}.

We then define a score for a candidate permutation vector *π *according to the scoring function , where



The permutation vector *π *is chosen to be a local minimum of *s*(*π*), which is found using the following local search procedure. Given a permutation vector *π*, let (*i*, *j*) denote the permutation vector resulting from swapping the *i*th and *j*th entries of *π*.

### Local search procedure

Step 1: Generate a random permutation vector *π*.

Step 2: Find (*i*, *j*) satisfying *s*((*i*, *j*)) <*s*(*π*). If no such (*i*, *j*) exists, stop; *π *is locally optimal.

Step 3: Set *π *= (*i*, *j*) and go to Step 2.

### Test results

To validate our algorithm, we performed three sets of tests on simulated data.

#### p-value tests

**Figure 1:**
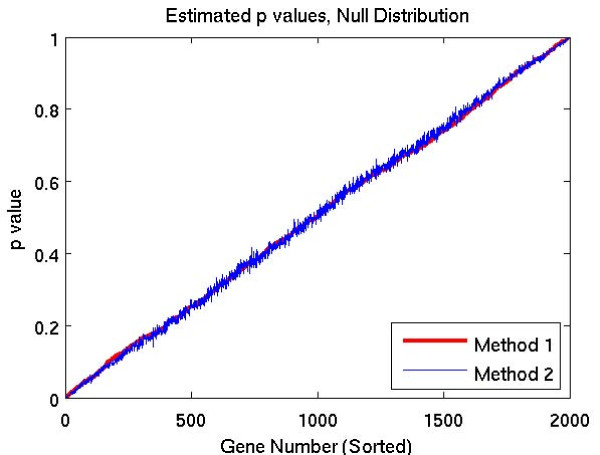
**Estimated p-values for null distribution**.

**Figure 2:**
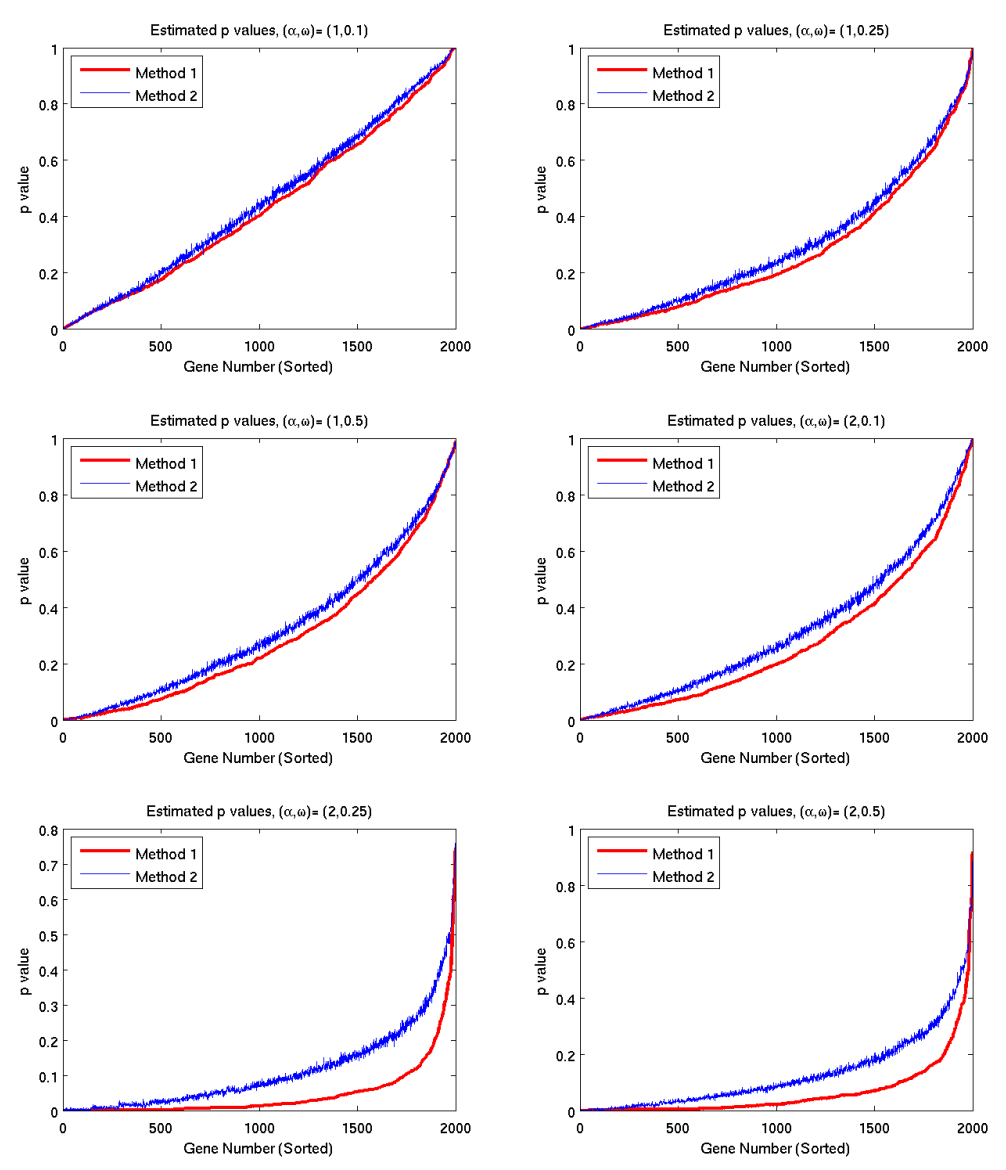
**Estimated p-values (low freq. signals)**.

**Figure 3:**
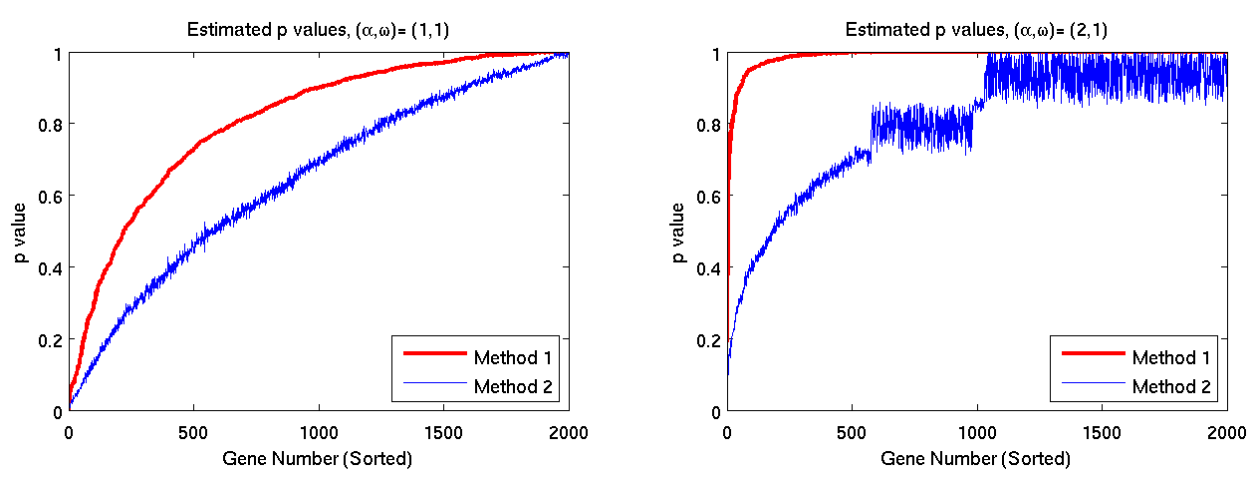
**Estimated p-values (high freq. signals)**.

In the first set of tests, we compared the p-values calculated by the two methods on 9 simulated data sets. Each data set corresponds to an underlying temporal variation with different frequency and amplitude, specified by parameters *ω *and *α*. (See (5) in Section 5.1 for details). The results are shown in Figures 1, 2, and 3. Figure 1 corresponds to the null case, where there are no temporal dependencies in the data. The graphs in Figure 2 correspond to cases where there is a temporal dependency whose frequency is relatively low, and the graphs in Figure 3 correspond to higher frequency temporal dependencies. In each graph, the simulated genes are sorted in order of increasing p-values for Method 1.

#### False discovery rate tests

**Figure 4:**
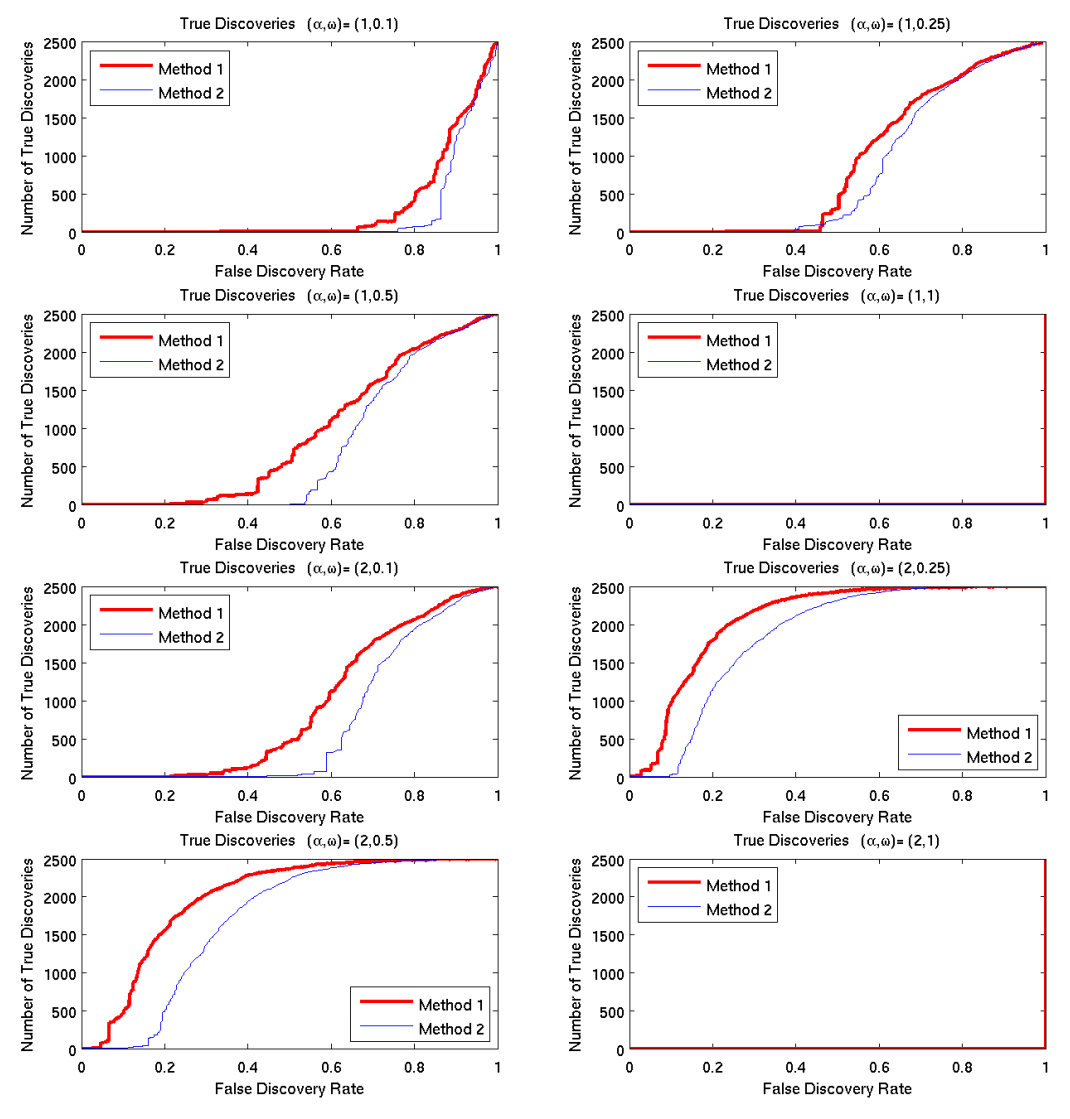
**True discoveries as function of fdr rates**.

The second set of tests analyzed 8 simulated data sets. In each data set, half of the genes were generated with time-dependent variation, and half were generated from the null distribution. For each method, we determined for each gene the smallest false discovery rate that would result in that gene being selected. By sorting the genes according to these threshold rates, we calculated the number of true discoveries and the number of false discoveries that would result from every possible fdr rate. The results are shown in Figure 4.

**Figure 5:**
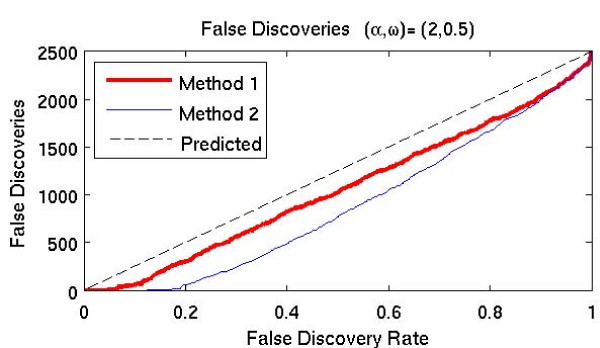
**False discoveries as function of fdr rates**.

We also plotted the number of false discoveries as a function of the specified fdr rate. An example for the case *α *= 2, *ω *= .5 is shown in Figure 5. In this figure, the dotted line corresponds to the predicted number of false discoveries for each false discovery rate. Observe that the true number of false discoveries is below this line for both algorithms. Results for the other cases are similar.

#### Sensitivity tests

Our third set of tests studied the sensitivity of the methods to i) the number of time points *T*, ii) the number of knots *M *+ 1, and iii) the order of the spline *K*. Each test analyzed a data set using different values of *T*, *M*, and *K*. (See Section 5.1 for details). The results are reported in Table 1.

**Table 1:** Average estimated *p*-values

Method 1, Order = 2					Method 2, Order = 2				
	R					R			

T	2	4	6	8	T	2	4	6	8

9	0.567	0.512	0.511	0.520	9	0.689	0.613	0.319	0.598

17	0.533	0.857	0.899	0.921	17	0.326	0.607	0.488	0.596

25	0.262	0.417	0.599	0.586	25	0.204	0.445	0.578	0.614

33	0.133	0.120	0.173	0.594	33	0.163	0.119	0.147	0.549

49	0.022	0.006	0.006	0.123	49	0.110	0.032	0.035	0.249

65	0.018	0.002	0.004	0.002	65	0.083	0.015	0.015	0.012

Method 1, Order = 3					Method 2, Order = 3				

	R					R			

T	2	4	6	8	T	2	4	6	8

9	0.657	0.566	0.483	0.475	9	0.688	0.643	0.450	0.602

17	0.443	0.870	0.819	0.874	17	0.284	0.639	0.403	0.616

25	0.143	0.201	0.575	0.610	25	0.218	0.211	0.593	0.584

33	0.121	0.090	0.494	0.587	33	0.183	0.096	0.385	0.540

49	0.020	0.006	0.006	0.014	49	0.120	0.035	0.028	0.052

65	0.014	0.003	0.002	0.001	65	0.082	0.019	0.011	0.007

Method 1, Order = 4					Method 2, Order = 4				

	R					R			

T	2	4	6	8	T	2	4	6	8

9	0.589	0.616	0.510	0.593	9	0.678	0.673	0.403	0.633

17	0.445	0.801	0.821	0.881	17	0.293	0.581	0.125	0.634

25	0.220	0.292	0.481	0.528	25	0.213	0.280	0.510	0.544

33	0.145	0.101	0.097	0.470	33	0.178	0.097	0.071	0.453

49	0.034	0.008	0.008	0.028	49	0.129	0.052	0.035	0.087

65	0.016	0.003	0.003	0.001	65	0.089	0.021	0.014	0.006

### Estrous cycle study

**Figure 6:**
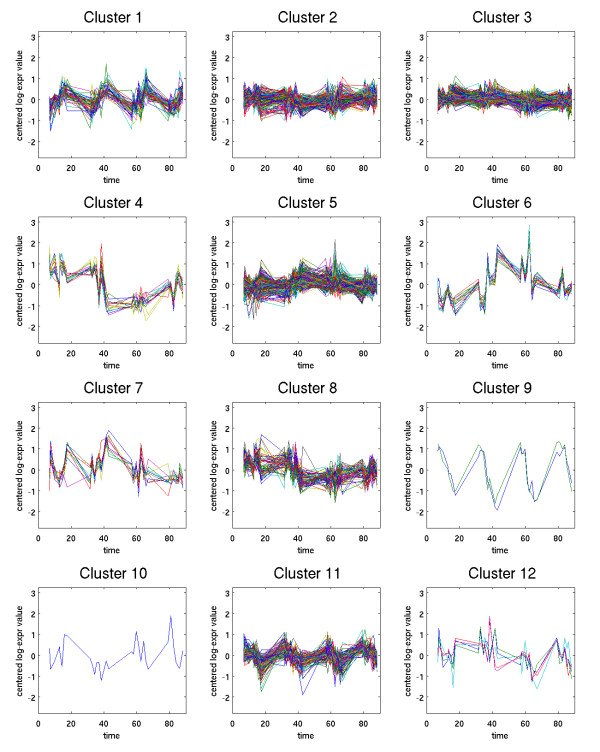
**Clusters 1–12**.

**Figure 7:**
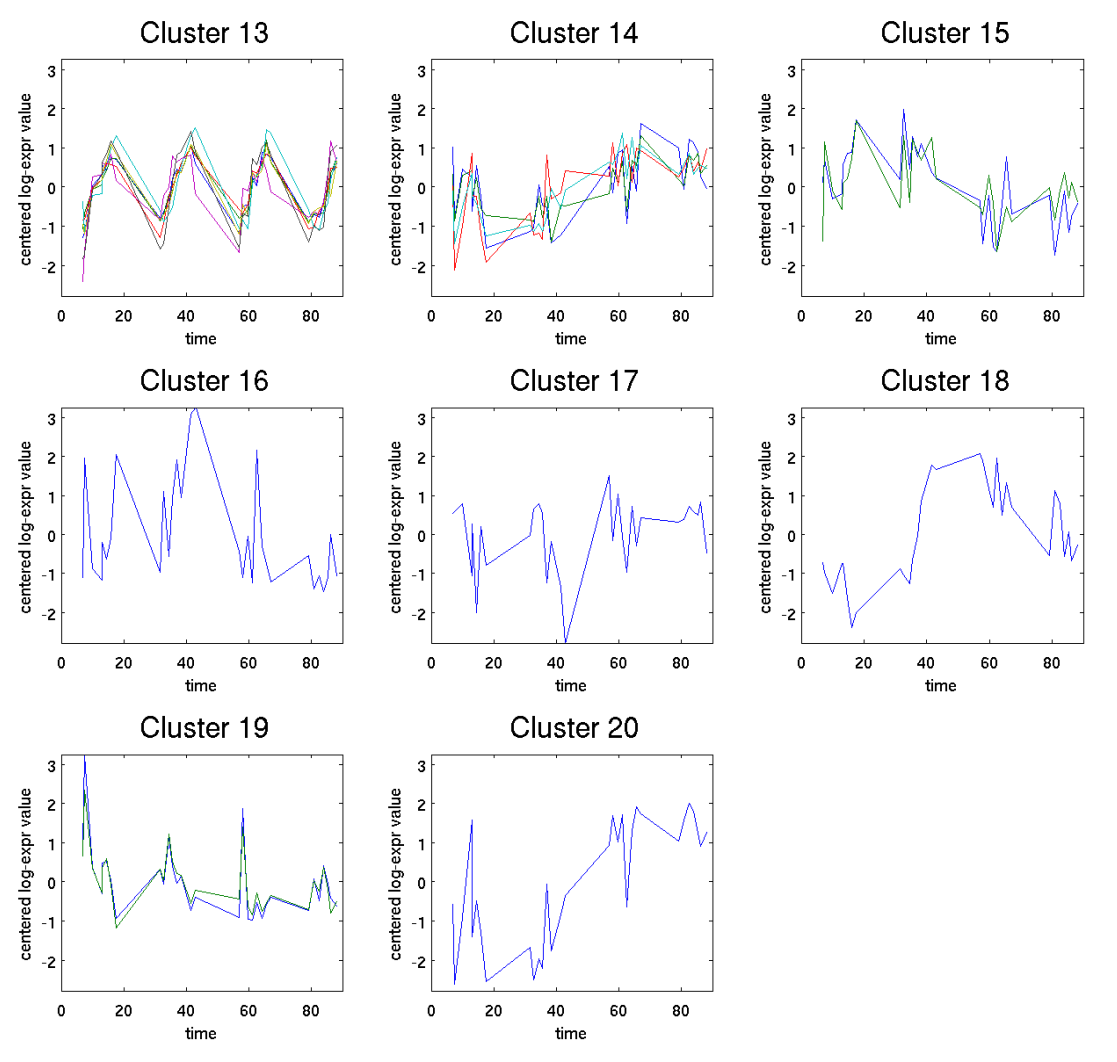
**Clusters 13–20**.

Method 1 was used to analyze a data set collected by microarray to study the estrous cycle of the virgin rat mammary gland. After preprocessing, this data set consists of expression levels of 21044 genes at 31 different time points, spread out over the 4 day estrous cycle. The application of Method 1 to this data set identified 1893 temporally significant genes. By comparison, Method 2 (using the same splines and fdr rate) identified only 871 genes. The 1893 genes identified by Method 1 were clustered using a hierarchical clustering method to generate 20 clusters, which are displayed in Figures 6 and 7.

The choice of clustering methods resulted in most of the genes (1579) being grouped into three large clusters (2,3 and 5). Examining the individual expression profiles of genes in these clusters shows that most of these genes exhibit relatively weak, but easily discernible low frequency variation. However, due to the large numbers of genes in these clusters, the graphs of the clusters (particularly clusters 2 and 3) do not exhibit obvious temporal patterns. While we believe that most of the genes in these clusters are temporally significant, it is also likely that these three clusters are richer in false positives.

**Figure 8:**
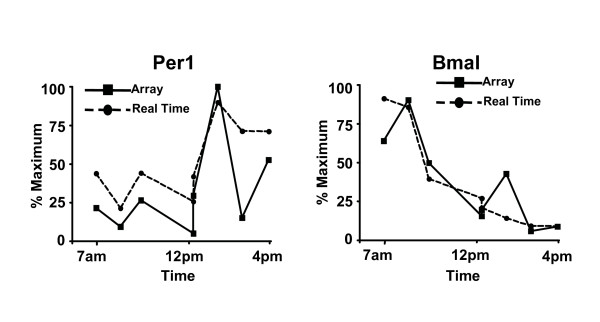
**Confirmation of Per1 and Bmal expression**. Total RNA from the indicated time-points was used for array analysis and analyzed by real time PCR using primers specific to rat Per1 and Bmal. Data was normalized to 100% of maximum and compared to that obtained in the arrays. The results show that Per1 and Bmal are under circadian regulation, validating the circadian patterns detected by analysis of the microarray data.

Nine of the clusters exhibit clear estrous-cycle dependent temporal responses. Clusters 4, 8, 12, and 15 are down-regulated immediately following Estrus (which occurs on day 2). Clusters 6, 7, 14, 18, and 20 are all up-regulated following Estrus. Four other clusters exhibit distinct circadian variation. In clusters 1 and 13, expression levels increase steadily throughout the day (7 a.m.–7 p.m.) and then decrease over night. The opposite behavior appears in clusters 9 and 11, for which expression levels decrease throughout the day. The presence of these circadian variations was unexpected and potentially quite significant biologically. To provide a preliminary validation of these results, two genes were selected for further study: Per1 (period homolog 1), which appears in cluster 1, and BMal (brain-muscle-ARNT-like protein), which is in cluster 9. For these two genes, quantitative real time PCR was performed, as described in [6]. The results of this analysis, shown in Figure 8, confirm that both genes are under circadian regulation and follow the patterns predicted by the clusters in which they are found.

## Discussion

### Frequency response

The results of the p-value tests shown in Figures 1, 2, and 3 demonstrate that the methods are sensitive to the frequency of the underlying temporal variation. Figure 1 corresponds to the null case (*α *= 0), where there are no temporal dependencies in the data. In this case, both methods yield essentially identical p-values, whose graphs lie on the 45 degree diagonal line. This line corresponds to the uniform distribution, which is the expected distribution of p-values arising from a null distribution. The graphs in Figure 2 correspond to cases where there is a temporal dependency whose frequency is relatively low. In these cases, the *p*-values of Method 1 are lower than the *p*-values of Method 2. As a result, Method 1 identifies a greater number of significant genes for any specified p-value. In contrast, the graphs in Figure 3 correspond to higher frequency temporal dependencies. In these cases, the p-values for Method 1 are higher, suggesting that Method 2 would be more sensitive. However, in these cases, the p-value distribution for both methods lies above the 45 degree diagonal line corresponding to the uniform distribution. Thus, when used in conjunction with a false discovery rate method, neither method would identify *any *of the genes as significant.

This phenomenon is surprising when viewed from the perspective of Method 2. That method was developed as a generalization of ANOVA techniques to the analysis of time series data. The test statistic is a straightforward generalization of the F statistic, and its null distribution is estimated using bootstrapping. From this perspective, one would expect that data containing time dependencies would, on average, produce lower p-values than the null distribution. But just the opposite is observed for high frequency data.

In contrast, the observed phenomenon is quite natural from the viewpoint of Method 1. Here, we are explicitly trying to measure the smoothing effect of the spline approximation on the data. When high frequency variation is present, the effect of smoothing will be large, resulting in larger p-values than one would expect from purely random data.

The impact of frequency on the calculated p-values is more exaggerated in Method 1. That is, for low frequency variation, Method 1 yields lower p-values than Method 2; and for high frequency variation, Method 1 yields higher p-values. Since neither method will detect high frequency variation, Method 1 appears to be more sensitive where it matters-i.e., in detecting low frequency variation.

This observation is confirmed by the results of the false discovery rate tests shown in Figures 4 and 5. Note that for any given false discovery rate, Method 1 detects a larger percentage of the time-dependent genes, with the exception of the high frequency cases, for which no genes were detected by either method.

### Sensitivity tests

The average estimated *p*-values reported in Table 1 give an indication of how well each method does in identifying temporally significant genes, with smaller values indicating better performance. We make the following observations:

1. Both methods are sensitive to the total number of knots. In particular, the methods perform best when there are at least 9 knots (which divide the range of times into 8 knot-intervals). For example, for *T *= 49, the *p*-values are very small for *R *= 4 (which corresponds to 13 knots) and *R *= 6 (9 knots), but get significantly worse for *R *= 8 (7 knots). This makes intuitive sense because the data were generated from a function which cycles 4 times, so 9 knots corresponds to two knot-intervals per cycle. Using fewer knots would make it very difficult to fit the data with the splines.

2. On the other hand, there can also be too many knots. For large numbers of time points (for example *T *= 65), the average *p*-values are worse for *R *= 2. This suggests that when there are relatively few time points per knot, the splines may be overfitting the data, resulting in poorer performance.

3. Both methods are relatively insensitive to the order of the splines.

### Estrous cycle study

In the estrous cycle study, it is interesting to note that all genes identified by Method 2 were also identified by Method 1. To assess whether the additional genes identified by Method 1 are biologically significant, we examined each of the 20 clusters and identified which genes in the cluster were also identified by Method 2. The results are shown in Table 2.

**Table 2:** Analysis of clusters

Cluster	# Method 1	# Method 2	# in Method 1 not Method 2	% in Method 1 not Method 2	Description
1	57	48	9	16	Circadian, Increase
2	318	147	171	54	No clear pattern
3	645	235	409	63	No clear pattern
4	14	14	0	0	Downreg. after E (PR in here)
5	611	287	324	53	No clear pattern
6	18	10	8	44	Upregulated after E (cyclins)
7	10	3	7	70	Upregulated after E
8	66	48	18	27	Downregulated after E
9	2	2	0	0	Circadian, decrease
10	1	0	1	100	No clear pattern
11	127	81	46	36	Circadian, decrease
12	5	2	3	60	Downregulated after E
13	7	7	0	0	Circadian, increase
14	4	4	0	0	Upregulated after E
15	2	1	1	50	Downregulated after E
16	1	1	0	0	No clear pattern
17	1	0	1	100	No clear pattern
18	1	1	0	0	Upregulated after E
19	2	0	0	0	No clear pattern
20	1	1	0	0	Upregulated after E

The genes found only by Method 1 are spread across most of the clusters (with only some of the very small clusters missing). As a general trend, Method 2 identified a larger percentage of genes for clusters with more distinct temporal patterns. For example, Method 2 identified all the genes in cluster 4, which has a very distinct pattern; whereas it identified less than half of the genes in clusters 2,3 and 5, which exhibit far less obvious temporal patterns. This suggests that many of the additional genes found by Method 1 may be false positives. However, there are some clear exceptions to this general trend. For example, Method 2 found only 56% of the genes in cluster 6, despite the fact that the genes in this cluster have nearly identical expression profiles, and exhibit a very distinct temporal pattern. Clearly, Method 1 is identifying biologically significant genes that were missed by Method 2.

## Conclusion

The algorithm presented in this paper provides a method for identifying significant temporal variation in time course gene expression data without requiring replicates. This experimental paradigm enables researchers to collect data at more time points, which often provides greater biological insight. This was evident in these experiments where the procedure allowed the discovery of significant circadian variation in a substantial number of genes.

Based on our simulation tests, when compared with the method described in [2], our method appears to be more sensitive to detecting low frequency (relative to the sampling rate) time-dependencies for any given false discovery rate. Neither method is effective at identifying higher frequency temporal trends.

Specifically, the results of the sensitivity tests indicate that to detect variation of a given frequency, at least 8 time points per cycle are needed.

The method was used in a study of the estrous cycle in rat mammary glands revealing the presence of genes with circadian variation. The circadian patterns for two genes, Per1 and Bmal, were validated by quantitative real time PCR analysis. Additional patterns have been validated in follow-up experiments. The identification of circadian genes by our method highlights the advantage of evaluating non-replicative time course data in comparison to replicate data. Specifically, if replicate and no-replicate studies had been undertaken with the same number of subjects (animals), the circadian patterns would not have been identified in the replicate study.

## Methods

### Simulation tests

The simulation tests were performed as follows. In the p-value and FDR tests, data were generated using time points *t *= (0, 2, 4, 6, 8, 10, 12, 14, 16, 18, 20), and the two methods were tested using a 4th order spline with knots [0, 0, 0, 0, 5, 10, 15, 20, 20, 20, 20].

In the p-value tests, we generated 9 data sets, each consisting of the log-expression values of 2000 genes generated from the following model, with timepoints *t *= (0, 2, 4, 6, 8, 10, 12, 14, 16, 18, 20):

(5) *Y*_*ij *_= *α *cos(*ωt*_*j*_) + *N *(0, 1).

Here, *α*, and *ω *are parameters that control the magnitude and frequency of the time dependent variation in the data, and *N*(0, 1) is the Gaussian distribution with mean 0 and variance 1.

The null data set was generated using *α *= 0. The other 8 data sets were generated using all combinations of the following parameter values *α *= 1 or 2, and *ω *= .1, .25, .5 and 1.

In the FDR tests, we generated 2500 time-dependent genes using Equation (5) and 2500 genes from the null distribution, for a combined data set of 5000 simulated genes. For each method, we determined for each gene the smallest fdr rate that would result in that gene being selected. By sorting the genes according to these threshold fdr rates, we could easily calculate the number of true discoveries and the number of false discoveries that would result from every possible fdr rate. These test were performed for the same 8 combinations of *α *and *ω *that were used in the p-value tests.

In the sensitivity tests, we generated 500 time-dependent genes from (5) with *α *= 1, *ω *= 1, with *T *evenly-spaced time points ranging from *t*_*min *_= 0 to *t*_*max *_= 20. With this choice of parameters, the cosine function in (5) cycles roughly 4 times over the range of time points. Both methods were run on the simulated data set using *M *+ 1 evenly spaced knots ranging from *t*_*min *_= 0 to *t*_*max *_= 20. For Method 1, the permutation vector *π *was chosen according to the procedure described in Section 2.1.3. For Method 2, a sample size of 500 was used for the bootstrapping procedure. For each run, we calculated the average of the estimated *p*-values for all genes in the data set. The tests were repeated for all combinations of *T *∈ {9, 17, 25, 33, 49, 65}, *K *= {2, 3, 4}, and *M *= (*T *- 1)*R *for *R ∈* {2, 4, 6, 8}. Note that the number of knots *M *+ 1 used in each case depends on the number of time points *T *and the ratio *R*. Specifically, *M *= (*T *- 1)/*R*. Thus, smaller values of *R *correspond to larger numbers of knots.

### Estrous cycle study

The data analyzed in the estrous cycle study were generated as follows. Sprague-Dawley female rats were obtained from Taconic Farms (Germantown, New York, USA) as adult virgins 70 +/- 3 days of age. Vaginal lavages were performed daily to identify rats with regular 4 day estrous cycles, as previously described [7]. Rats were included in the study after confirmation of at least two consecutive and regular 4-day estrous cycles. Cervical tissue, harvested and prepared for histological analysis, was used to confirm stage of estrous at time of euthanasia. Collecting each tissue sample required sacrificing the rat, so each microarray measured expression levels from a different rat. This resulted in significant heterogeneity between samples that was independent of time. Rats were sacrificed at approximately 1.5 hour intervals between 7 am and 7 pm over the four day estrous cycle. Blood samples were taken for hormone levels and a mammary gland was dissected and flash frozen to be used for later mRNA extraction. Left mammary gland chains 4–6, with lymph nodes removed, were quick frozen in liquid nitrogen for biochemical and molecular analyses. All animal procedures were done in compliance with the AMC Cancer Research Institute Animal Care and Use Committee and NIH Policy on Humane Care and Use of Laboratory Animals. Total RNA was isolated from approximately 100 mg fractions of frozen, pulverized mammary tissues using TriZol reagent as per the manufacturer's supplied protocol (Invitrogen Life Technologies, Inc, Carlsbad, California, USA). RNA samples were further purified and DNase treated using RNeasy and the RNase-Free DNase Set (Qiagen, Valencia, California, USA) as per the manufacturer's suggestions. Quality and quantity of total RNA was assessed using the RNA 6000 Nano Assay (Agilent Technologies, Palo Alto, California, USA). First and second strand cDNA synthesis was carried out using a one-cycle method (first strand then second strand synthesis) employing an Invitrogen cDNA synthesis kit as outlined in the Affymetrix 2003 protocol. The double stranded cDNA product was cleaned up and used as template for target labeling in vitro transcription reaction (Affymetrix GeneChip IVT Labeling kit). Twenty *μ*g of amplified IVT product was fragmented, and the quality of both the IVT product and the fragmentation product were assessed using the Agilent Bioanalyzer system. All samples passed and were subsequently hybridized to the Rat RAE_230 2.0 Affymetrix microarray chips. Hybridized chips were scanned, data collected and scaled to a target gene intensity of 175 using GeneChip Operating Software™ (GCOS) version 1.1 (Affymetrix, ). Initial quality assessment of all scanned chips was performed using GeneChip Operating Software (GCOS) v1.1. Compiled data in the form of 32 individual CEL files, the primary output of scanned Rat RAE_230 2.0 microarray chips, were imported to GeneSpring (Agilent Technologies, ) for analysis using the native probe level GC-Robust Multi-array Average (GC-RMA) algorithm. Incomplete and ambiguous data was discarded leaving samples at the following 31 time points (measured in hours after midnight of the first day of the estrous cycle): 7, 7.5, 10, 13, 13.2, 14.6, 16, 17.6, 31.6, 32.8, 34.5, 35.5, 37.1, 38.3, 41.6, 43, 57, 58, 59.8, 61.3, 62.5, 64.2, 65.6, 67.1, 79.3, 80.9, 82.5, 83.9, 85.3, 86.3, 88.1. (Time point 40 was discarded because the data appeared corrupted). After processing, consensus expression levels were available for each gene at each time point. In order to minimize the possible adverse impact of low-level noise on the analysis, any gene that had a consensus expression level reading of less than 10 at any time point was deleted. This procedure resulted in a data set consisting of 21044 genes at 31 time points. Raw data are deposited in the National Center for Biotechnology Information Gene Expression Omnibus (GEO series GSE12289). Prior to applying our algorithm, we performed the following normalization procedure to the expression values:

*Y*_*ij *_= log *X*_*ij *_- *μ*_*i*_,

where *X*_*ij *_is the consensus expression level for gene *i *at the *j*th time point, and *μ*_*i *_is the average value of log *X*_*ij *_for the *i*th gene.

The algorithm was applied to the normalized data *Y*_*ij*_, using a 2nd order (piecewise linear) spline defined with the following knots sequence: 7,7,17.6,31.6,43,58,67,79.3,88.1,88.1. These knots correspond to the first and last time point collected for each day.

The null distribution of the test statistic was calculated using the permutation vector *π *= (7, 14, 23, 30, 1, 15, 20, 26, 6, 9, 19, 29, 3, 10, 22, 28, 2, 13, 24, 5, 11, 25, 27, 8, 17, 21, 31, 4, 12, 18, 16).

Significant genes were identified using a false discovery rate of *f *= .25. The genes were clustered by applying a hierarchical clustering method to the spline coefficients calculated by equation (3). A complete linkage method was used, and the distance between two genes *i *and *j *was defined using the Euclidean distance . From this, 20 clusters were identified.

In the validation study, two genes were selected for further study: Per1 (period homolog 1), and BMal (brain-muscle-ARNT-like protein). For these two genes, quantitative real time PCR was performed, as described in [6]. Primers used for analysis were: Bmal-F1 5'-GGG CTG GAC GAA GAC AGT GA-3', Bmal-R1 5'-CGC CCG ATT GCA ACG A-3', Per1-F1 5'-CCT GCA CAC CCA GAA GGA A-3', Per1-R1 5'-GAG GTG TCA AGC CCA CGA A-3', Actin-F1 5'-TCT GTG TGG ATT GGT GGC TCT A-3', Actin-R1 5'-CTG CTT GCT GAT CCA CAT CTG-3'.

## Authors' contributions

SB and MN contributed the initial concept for this work and obtained funding for the experimental work. PS supervised the rat experiments and MR performed the RNA extraction, Affymetrix benchwork and initial microarray analysis using GeneSpring. Weston Porter's lab performed the quantitative real time PCR analysis of the Per1 and Bmal genes. SB contributed the major mathematical analysis and the paper was written with additional insights from the other authors. All authors read and approved the final manuscript.

## References

[B1] Bar-Joseph Z (2004). Analyzing time series gene expression data. Bioinformatics.

[B2] Storey JD, Xiao W, Leek JT, Dai JY, Tompkins RG, Davis RW (2005). Significance Analysis of Time Course Microarray Experiments. Proceedings of the National Academy of Sciences of the United States of America.

[B3] de Boor C (1978). A Practical Guide to Splines.

[B4] Golub GH, Loan CFV (1983). Matrix Computations.

[B5] Benjamini Y, Hochberg Y (1995). Controlling the false discovery rate: A practical and powerful approach to multiple testing. Journal of the Royal Statistical Society B.

[B6] Metz RP, Qu X, Earnest D, Porter W (2006). Circadian Clock and Cell Cycle Gene Expression In Mouse Mammary Epithelial Cells and in the Developing Mouse Mammary Gland. Dev Dyn.

[B7] Schedin P, Mitrenga T, Kaeck M (2000). Estrous cycle regulation of mammary epithelial cell proliferation, differentiation, and death in the Sprague-Dawley rat: a model for investigating the role of estrous cycling in mammary carcinogenesis. J Mammary Gland Biol Neoplasia.

